# Some Chemical Problems as Applied to Dentistry

**Published:** 1922-09

**Authors:** Albert K. Epstein


### SOME CHEMICAL PROBLEMS AS APPLIED TO DENTISTRY

BY ALBERT K. EPSTEIN

*Read before the meeting of the Odontological Society of Chicago, Ill., January 5, 1922 and printed in "Dental Cosmos," June 192?.

Chemistry has contributed very much to the medical sciences and also to applied dentistry. It will be impossible for me to survey the entire field of chemical problems which are and which will be of service to the dental profession. I shall not speak about those branches of chemistry, such as ceramics, metallurgy, the chemistry of the vulcanization of rubber, or the chemistry of local anesthetics, which have been applied to dental practice.

I wish to speak particularly of two specific problems: First, the chemistry of commercial mouth preparations; second, the importance of the diet as related to decalcification of teeth. The latter problem is not only interesting to dentists from a scientific and therapeutic standpoint, but also to the ordinary layman. American biochemists have contributed considerable in the past ten years to this branch of nutritional chemistry.

The saliva can be looked upon as changed protoplasm. As we all know, it contains a large amount of moisture, approximately 99.5 per cent., and about .5 per cent. of organic materials, such as mucin, epithelial cells, and also inorganic matter, such as calcium, phosphates, chlorids, etc. It is interesting to note that of the nine elements which are important to sustain life, seven are found in the saliva. The saliva in a healthy mouth is slightly alkaline.

In the newspapers and in the advertisements accompanying commercial mouth preparations, such as toothpastes and mouthwashes, we find considerable discussion regarding the alkalinity and acidity of the mouth, and also recommendations to the public to use an acid or alkaline toothpaste, depending on whether the manufacturers happen to place on the market an acid or alkaline preparation. If we survey the early literature regarding this subject we find widespread confusion with reference to the reaction of the saliva. A number of authorities advocate the use of alkaline mouth preparations, while others advocate the use of acid preparations. Even conclusions derived from experimental observations seem to contradict each other. There are statements that the teeth are soluble in weak dilute organic acids, such as lactic acid, while there are other statements to the contrary.

This appearent confusion results from the fact that our old idea of alkalinity and acidity was not definite in character. It was customary to designate acidity qualitatively by the change of color obtained by the use of a certain dye or indicator. The degree of acidity was designated quantitatively by the titration method, using an indicator as an endpoint. But saliva is alkaline to litmus paper and will give an acid reaction if you use phenolphthalein as an indicator. The question arises, therefore, is there a definite line of demarcation which will tell us the exact neutral point irrespective of a particular dye or indicator which is used, and irrespective of the substances which are present in solution.

The conception of the electrolytic dissociation of matter, which has been under academic study for many years, has furnished us a definite method by which we can measure the degree of acidity and alkalinity of a solution. This method has helped to solve many problems in biological sciences and will play an important part in solving and clearing up confusions in problems relating to dentistry. The method consists in determining the hydrogen-ion concentration of a fluid.

It is known that pure distilled water ionizes into hydrogen ions, or hydrogen carrying a positive charge of electricity, and hydroxyl ions, or the OH group, carrying a negative charge of electricity. It is known that pure distilled water contains one-ten millionth of a gram of hyrogen ion per liter and one-ten millionth of a gram of hydroxyl ion per liter, and, therefore, it has a neutral reaction. If we add some substance to the water solution which will increase the hydrogen ion, for instance, to one-hundred thousandth of a gram of hydrogen ion or (H) $10^{-5}$, then the solution will be acid. It the hydrogen ion is increased to $10^{-4}$ per liter it will be still more acid. On the other hand, if a substance is added to the water solution which gives off OH ions so as to decrease the hydrogen-ion concentration to one-hundred millionth or $10^{-8}$, the solution will become alkaline.

The essential characteristic of an acid is to give off hydrogen ions in water solution. The different degree to which the hydrogen ions are liberated determines the relative strength of an acid. For example, one-tenth normal hydrochloric and lactic acids both contain one-tenth of a gram of replaceable hydrogen per liter, but hydrochloric acid will give off more hydrogen ions or is more dissociated and, therefore, will be stronger than lactic. The "titratable acidity" of equal quantities of the two solutions will be the same and it will take the same amount of sodium hydroxid to neutralize them, if a suitable indicator is used. However, the "actual acidity" is different in the two solutions, as they both contain different amounts of hydrogen carrying a positive charge.

We, therefore, designate a solution containing hydrogen-ion concentration of $10^{-7}$, or as Sorensen designates it "Ph 7," as neutral. If the solution contains hydrogen ions less than this quantity, for instance, such as (H) $10^{-8}$ or Ph 8, it is alkaline. If the solution contains hydrogen ions greater than (H) $10^{-7}$ or Ph 7, then it is acid.

The indicators used in titration methods to determine the degree are nothing else than certain organic coloring substances, which have the properties of weak acids and bases which undergo a definite change of their structure at a definite hydrogen-ion concentration, accompanied by a change of color. Methylorange changes its color at a hydrogen-ion concentration to $10^{-4}$, while phenolphthalein changes its color at a hydrogen-ion concentration to $10^{-9}$, which is on the alkaline side. We can readily see that titration methods by means of indicators do not give us the "actual acidity" of a solution.

Furthermore, saliva contains certain substances which are known as "buffers," such as protein material, bicarbonates and phosphates. Although these substances are titratable and will unite with alkalies and acids, however, the addition of small amounts of acids and alkalies to these buffer substances present in saliva will have only a very slight effect upon the change of hydrogen-ion concentration. We can readily see, therefore, why there are so much confusion and contradictory statements regarding the reaction of the saliva and the effect of acids and alkalies on teeth. The modern methods of the determination of hydrogen-ion concentration help to clear up this confusion and also help to explain puzzling phenomena and will help to solve the problem of acidity or alkalinity of commercial mouth preparations.

The calcium salts which are present in saliva are in a solution from which they may be precipitated by the change of the hydrogen-ion concentration. An increase of ammonia in the breath or a change of the carbon-dioxid tension in the blood will have a tendency to change the hydrogen-ion concentration of the fluids of the mouth. This change will cause the precipitation of the calcium in the form of phosphates and carbonates. This precipitate embedded in organic matter as epithelial cells and the débris of food forms a layer on the teeth known as tartar.

If this layer is not very heavy, toothpastes and mouthwashes are recommended to remove it. The active principle of some toothpastes and mouthwashes is a substance of an alkaline or acid nature which dissolves one or the other ingredient of the tartar. If an acid substance is used the acid will dissolve out the calcium phosphate, thereby loosening the tartar. If an alkaline substance is used the organic protein material will be dissolved. Experimental facts show that some alkalies have a bad effect upon the teeth, while strong acids may injure the enamel.

The advertising managers who advocate in pseudo-scientific phraseology one or the other mouth preparation have not the slightest idea regarding the intricacies of these problems, and are taking advantage of the confusion existing in the literature and often misrepresent scientific facts. Only on rare occasions does the manufacturer have his product investigated from a scientific standpoint. A popular tooth-paste consists of calcium phosphate or precipitated bone, calcium sulphate, a small amount of pepsin and hydrochloric acid mixed with a glycerin-water solution. The manufacturers originally claimed that the pepsin would digest the organic materials in the tartar, and thereby loosen it. It is known that pepsin, which is an enzyme, acts at a certain rate. A definite amount of enzyme will digest a definite amount of protein material at a definite temperature and a definite hydrogen-ion concentration. It must also be remembered that dispersion has a great effect upon the action of enzymes and that the action of pepsin in a liquid is different from that in a heavy paste. The question arises, therefore, will the pepsin be able to digest the tartar in the form in which it is dispersed at the short interval of time during which it is applied in the mouth? Will the relatively high hydrogen-ion concentration, which is favorable to the enzyme action, injure the enamel of the teeth if the paste is used constantly. Usually the advertising managers do not bother themselves to find out experimental facts as long as they have some scientific talking points which sound well.

There are a number of other advertised toothpastes on the market which have magnesium oxid as a base, although there are some authorities who state that this substance is not good for the teeth. There are some authorities in the dental literature who claim that soap softens the gums, but there are advertising managers who claim to know better and recommend such toothpastes to the public.

We can readily see that the problems involved in the use of the proper mouth preparations are too complex to leave them to the lay advertising writer to solve. The American Medical Association with the aid of their chemical experts have done splendid work in taking off the market fictitious remedies and also suppressing fraudulent and exaggerated claims regarding medicines. The dental profession ought to follow the same example and establish a research institution where the various preparations on the market should be investigated from a chemical and therapeutic standpoint.

### VITAMINS.

The other problem which I want to touch on is the question of vitamins. Newspaper advertisements are informing us that if we buy vitamins in the form of some tablets, we will lose all our ailments and live happily.

Many of the manufacturers of these preparations have taken advantage of scientific facts for personal gain. However, there is something about vitamins which is of importance and of great value. In the olden days we were taught that a perfect diet ought to contain a certain amount of calories or heat units. It was discovered subsequently that the source of the calories is important and that they must be distributed in definite proportion among the three classes of foods—carbohydrates, protein material and fats.

A third step in the development of the science of nutrition is the discovery of the fact that not all proteins are equally beneficial to us. Proteins are composed of so-called amino-acids. There are eighteen amino-acids now known and protein contains all the amino-acids in a definite proportion. It would be impossible for a human being to remain in good health if he were to derive his nitrogen from gelatin only. Gelatin is not a complete protein. It lacks a few of the amino-acids, such as tyrosin and tryptophan.

It was also discovered that we must have in our diet a certain amount of minerals of the following nine elements: Calcium, magnesium, sodium, potassium, phosphorus, iron, sulphur, iodin and cholrin. If in our diet any one of these elements is absent or not supplied in sufficient quantity we will have a metabolic disturbance resulting in some sort of ailment.

In nutrition experiments performed on animals, it was discovered that if an animal was given a diet consisting of purified casein as a source of nitrogen, purified starch as a source of carbohydrates, olive oil as a source of fats and the necessary mineral elements, such animals would not grow on such a diet. Growth will be stunted and the animal will develop certain ailments. If green vegetables or butter fat is added to such a diet or an extract of butter fat or of green vegetables, then the animal will recover and in course of time will regain its normal weight.

These substances which are present in some natural foods, but not in refined food, are called "vitamins" or "food accessories." Their exact chemical nature is not known, although there are at present considerable experimental data regarding the stability of these vitamins toward heat and their behavior toward various solvents and other chemical reagents.

Professor McCollum of Johns Hopkins Hospital and his associates are pioneers in this branch of nutritional chemistry.

At present three different kinds of vitamins are known. From a biological standpoint, they are recognized by different specific diseases which develop in an animal fed on a diet lacking one or the other vitamins.

"Fat-soluble A" vitamin is usually found in butter fat, milk, cream, eggs, cod liver oil, glandular organs of animals, and its absence in a diet is associated with eye disease and rickets.

"Water-soluble B" is usually found in yeast, milk and green vegetables, such as spinach, celery, alfalfa, lettuce, cauliflower, cabbage, etc. Its absence in the diet is responsible for the disease beriberi and polyneuritis in pigeons.

"Water-soluble C" is usually found in small quantities in milk and in larger quantities in vegetables, tomatoes, orange and other fruit juices. Its absence in the diet is associated with the development of scurvy.

It has been recently discovered that these vitamins are responsible for the proper assimilation of calcium. The absence of vitamins in food products produces a disturbance in the assimilation of calcium, although the diet may contain a sufficient quantity of this element. You can readily see the importance of this question and its bearing on decalification of teeth.

Experiments have been performed at Harvard University by Howe and others on feeding animals a diet containing all the necessary mineral consistuents and the proper amount of carbohydrates, proteins and fats, but which lacked some of these vitamins. The results were that the teeth of the animals were found to be loose, and some of them could be penetrated slightly with a sharp instrument. Inflammation resulted and degenerative tissue was noticed. An addition of vitamin to the diet resulted in a quick recovery. The Eskimo does not use a toothbrush, but it is claimed that the Eskimos have healthy teeth. This is explained by the fact that the Eskimo not only eats the flesh of the animals, but also the glandular organs, which contain the vitamins which are responsible for the proper assimilation of calcium. Calcium is important in the diet of adults as well as in that of the young.

Many of my friends who practice dentistry in foreign neighborhoods have informed me that the teeth of their patients, who have come recently from the war-stricken zone are in bad condition. On investigation it was found that they have lived for a long period of time on a restricted diet which was poor in vitamins. Which of the three known vitamins is responsible for the assimilation of calcium is at present a matter of scientific controversy, which may be cleared up in the near future by the scientists who are working in this field of research.

25 E. Washington St., Chicago, Ill.

(See also *Discussion,* as reported in June *Cosmos.*)

Discussion.

Dr. J. E. Hinkins. My attention was first called to diet fifteen or twenty years ago by Dr. Hopkins, of the Agricultural Department of the University of Illinois, in the feeding of dairy cattle and the methods of fattening the cattle they brought to the fat stock show. We found it was impossible to produce the fat cattle without giving them plenty of milk. We also found, at the same time, if in trying to fatten these animals we did not give a milk diet, it did not make any difference what else we gave them, we did not produce growth, and many times we produced health without knowing its cause.

I do not know very much about diet. Personally, I have been a great believer in a mixed diet. I eat many green vegetables and vegetable soups. I drink a great deal of milk, and I attribute my good health to my method of living. I am a pretty well-preserved man physically, considering that I am sixty-three years of age, and I attribute it largely to my diet. I have never been a great meat eater or of carbohydrates, starch or sugar, but I eat a mixed diet; I drink largely of water and buttermilk.

Twenty-five years ago Dr. Acrea and I did a lot of work trying to find out the number of organic acids in the saliva. We began this work at the University of Chicago and finished it at the Johns Hopkins. While working with the different enzymes in the mouth we found that hydrolysis of triacetyl glucose by enzymes was continually produced. We also found the action was reversible. At that time I was interested in some financial problems, and I had considerable erosion of the teeth. My saliva became quite acid, and for a year I would titrate 10 cc. of saliva every night at 5.30 against phenolphthalein and potassium hydrate, 1 per cent. normal. We found my saliva was one-sixth of one per cent. acid or near that. After considerable work along this line we came to the conclusion that with our chemical knowledge we had to find a definite line of demarcation between the acidity and alkalinity of the mouth, but we could not do it, and as I did not have the money to carry on the experiments the work was stopped. I cannot help but feel that Dr. Epstein, through his work, has solved a problem that we had worked at for years and were not able to find its solution, and I congratulate him on the good work he is doing.

To me it seems the progress of denistry must come through the chemical laboratory or some other branch that pertains to pathology, biology or physiology. I have made the statement in this Society before, and I repeat it tonight, some of you present will live long enough to see that bacteria are not half as bad to us as is the protein decomposition of end-products that results from biochemical action.

Dr. W. J. Carlsen. The only significant thing I can think of in connection with Dr. Epstein's paper is the possible connection between the drinking of milk and the assimilation of calcium salts and their proper distribution throughout the body and the possible connection between them and the stimulation of the thyroid gland secretion. Some of the English observers interested in endocrine gland work have done considerable research along that line, and it has been very conclusive that the drinking of pure milk and whole milk has a desirable effect upon the proper working of the thyroid gland. They have pretty definitely determined that the thyroid gland is responsible for the proper distribution of the calcium salts in the tissues of the body and bone, and in all cases practically of severe decay in the mouth and those of carious bone we are able to detect a faulty mechanism in the thyroid gland. I

would suggest to Dr. Epstein that, perhaps in his work as a chemist, he go farther into that one branch of bio-chemistry of the thyroid gland to see whether or not the drinking of milk and the assimilation of vitamins from pure fruit juices does not have some effect upon the body mechanism, and perhaps may stop the enormous amount of decay that we see in some cases.

Dr. Lester C. Bryant. I have not given much thought to the subject of vitamins, although we do know that foods play a large part in the decay of our teeth. I remember when Dr. G. V. Black brought out his theory of tartar forming on teeth and said he could control it by a light or a heavy meal, I did some experimental work along that line and I found that if at night I put the cover-glass of a tube on my first molar after eating a heavy meal, it would be coated in the morning with a deposit. If I ate a light meal, examining it in the morning, it would be found comparatively clean and with hardly enough deposit to take a stain. I did not seem to burn up what I had eaten when a heavy meal was taken. That is the way it appealed to me. The food not fully assimilated was thrown off as waste products. I always got a deposit after eating a heavy meal; I could stain the glass positively, whereas after a light meal I would have only a slight stain.

I was interested in Dr. Epstein's discussion of dentifrices and toothpastes. I think many of our pastes and dentifrices and so-called mouthwashes do a great deal of harm when indiscriminately used as laymen use them. Many of our mouthwashes are strong in drugs and our patients use them too strong, and in these strong solutions there are protoplasmic poisons which break down the tissues of the mouth. This is a slow, insidious breaking down of tissue. I recall a young man who came in the other day whose mouth I had regarded as a model because he had been properly taught how to clean the teeth. He came in with the gum tissue commencing to recede from the necks of the teeth on the lingual, buccal and labial surfaces. There was as much recession of the gum tissue on the lingual as on the labial surfaces. I said to him, "What have you been doing?" He replied, "I have been using a certain liquid preparation in cleansing my gums and teeth." There is no doubt in my mind that the agent he was using was responsible for that recession of the gums.

As the essayist has suggested, the dental practitioner ought to investigate some of these much-advertised mouth-washes and dentifrices and find out what is in them.

My experience in teaching patients how to brush the teeth and how to take care of their mouths, using a paste or powder as a lubricant, is to get stimulation by strenuous brushing. If they will spend the time in brushing their teeth to get the blood circulating properly through the tissues, I am not afraid of anything taking place so far as the breaking down of tissue is concerned. Fermentation goes on between the teeth because there is not time enough consumed in brushing the teeth, and I would rather have a patient brush his teeth twice a day thoroughly than a dozen times a day simply sweeping over the surfaces of the teeth.

Dr. C. N. Johnson. I wish to express my appreciation to Dr. Epstein for his interesting contribution. Regarding the investigations of Dr. G. V. Black, which were mentioned by Dr. Bryant, concerning the deposition of salivary calculus, I remember following Dr. Black in those investigations very closely. I had quite a number of discussions with him and went into the matter very carefully at the time. The summing up of his conclusion was that these deposits were formed largely as the result of overeating. He claimed, as Dr. Bryant has done, that he could deposit salivary calculus on a cover-glass on an artificial denture he wore in the upper jaw; that he could regulate that quite accurately by the amount he ate of certain foods. I had a patient in whose mouth salivary calculus was formed more rapidly than in any other I have ever known. I could clean the teeth as carefully as possible and in a week's time, with the ordinary care she gave them, there would be a definite deposit formed again. I inquired regarding her diet and found that for some months she had been on a diminished diet and was eating very little of anything. I therefore failed to demonstrate clinically the findings of Dr. Black in his work, and I know of no man who did his work more thoroughly and scientifically than did Dr. Black. It has appealed to me on many occasions that we do not find things turning out in a practical way the same as we find them demonstrated in the laboratory, and this is no criticism of laboratory work.

There is another thing in connection with the matter of diet which should claim the attention of not only the dental but the medical profession. I believe the human race has erred more in its diet, and particularly the American people, than in any other one thing. But this is apparent: What is a good nourishing and proper diet for one individual is not necessarily so for another. The personal equation enters into that. You cannot talk with people and study their diet without being impressed with it. We cannot settle that complicated question by rule of thumb.

Dr. G. Walter Dittmar. I was hoping that the essayist would also consider another subject which is far more interesting to us than salivary calculus, and that is, serumal calculus. Serumal calculus does very much more harm than does salivary calculus, and I hope the essayist will at some future time devote attention to this subject. Salivary calculus, if allowed to accumulate, does harm, but it is insignificant compared to the other form of calculus. What may cause it, and how it may be prevented are things which are interesting to us, because it does a great deal of harm to the supporting structures of teeth.

The subject of diet is very interesting, as is also that of the ductless glands. I suppose the essayist might answer a number of questions touched on by Dr. Johnson. For instance, why are some people fat and others thin, some large and others small even in the same family? These things, no doubt, are controlled to a great extent by the ductless glands.

Dr. L. L. Davis. There are more things to be discovered by biological chemists than have been given us at the present time. The theory of the ductless glands has been known for a long time, yet it has been held in abeyance, laughed at, frowned down, and is now coming to the front, is being taken up and thoroughly studied by a number of good men, who are claiming considerable benefit from this study. There is certainly something to this balance of gland secretion. For instance, the adrenals against the thyroid, or the thyroid against the pancreatic, and so on. If we have a disturbance of any one of these glands they may inhibit or excite the other glands to function improperly, and so the thing is not to find out which is the gland that is functioning improperly, but to find out the gland that is upsetting the balance of the other.

There is one thing the essayist touched too lightly on, and that is the subject of dentifrices. I wish Dr. Sidney Knowles could have been with us this evening, because I would like to pay tribute to his uncle, Dr. Wassall, who happened to be, like myself, just new to the profession in Chicago in 1884. He and I worked together for many years, and in what little I have done along the line of cleansing the teeth I have followed out Dr. Wassall's theory. As early as 1885 I can remember Dr. Wassall talking about throwing aside all dentifrices and depending on clear water and "elbow grease" to clean the teeth, as he put it. Time and again in the meetings of this Society he emphasized that point, and at one time he gave each member of this Society one of these five-minute egg-timers. At that time it was his custom to give each one of his patients one of these egg-timers and insist upon them brushing the teeth during the time the sand was passing from one receptacle to the other. If we would emphasize the necessity for more thorough brushing and decry the use of these mouthwashes and various toothpastes, I think probably patients would be improved in the general health of the mouth. Any habit that is persisted in for any great length of time will act injuriously. There is no doubt in my mind that the use of the medicaments in these dentifrices must play an important part upon the tissues on which they are used, and so if one is in the habit of using a dentifrice that contains a quantity of a drug that is so cooling or stimulating to the mucous tissues of the mouth after its use, if kept up long enough, there will be a reaction against that kind of drug; therefore, I think we should instruct our patients to abstain from anything which tend to increase the drug habit. A patient can certainly get into the drug habit by the use of dentifrices. The dentist should know when to prescribe a mouthwash or cream or powder, and it should be for a recognized purpose, containing the drug necessary to the case, because there is no such thing as an all-round "cure-all" for mouth conditions, despite the claims of the various manufacturers.

Dr. Truman W. Brophy. The question of diet, which pertains not only to the dental patient, but to all patients and to all people, whether patients or not, is of the utmost importance. Of all the subjects we have to deal with, I think diet is the one that is the most disregarded, and consequently we suffer more from not clearly understanding and properly carrying out the uses of suitable foods than anything else. It would seem mankind through the centuries would have developed a diet suitable for everybody, but it has not been done. Chemists are working assiduously with the object in view to determine the kind of diet that is essential to people in the various conditions, physically and by environment, that will be suitable for them. Dr. Johnson wisely said that we cannot arrange a diet for one individual and say that is a diet for everybody. We know it will not do, and that is why chemical laboratories must be used more extensively than they have been in working out these questions of diet. Regarding the teeth, when there is either dissolution or the teeth are going wrong, the gum receding, as has been said by Dr. Bryant, by reason of certain preparations that are used, it is the business of the professional man to take charge of these people that come to him, and tell them what they need and what they must do to avoid getting into an unfortunate condition.

Every scientific man wants an opening for his findings, so that they can be spread to the people indirectly. If such a man did not have such an opening, what would be the use of scientific research for the benefit of others?

Dr. F. E. Roach. We are very glad from our point of view that Dr. Epstein is taking some interest in the things that are of such vital importance of our profession. These are things that will be helpful to us and through us will be helpful to the people. The thought that he has suggested with reference to the accumulation of salivary deposits on teeth leads us to believe and know that he is giving this subject much scientific consideration, which will no doubt result in something definite for us in the future. We feel that there is much yet to be learned with reference to the means of preventing pathological conditions of all kinds in the mouth, and the thoughts given us with reference to tooth preparations, mouthwashes, pastes and powders, are very pertinent at this time. Those of us who have given these questions any thought at all recognize the fact that there is entirely too much use of mouthwashes and toothpastes of all kinds; that our patients would be much better off if they left these things alone. We are having to combat these things all the time. It seems the public has gotten a mania for using all kinds of mouthwashes on account of the cooling feeling and pleasant taste they have in the mouth, when we know that many of them are detrimental rather than beneficial. We should impress upon our patients that these preparations, particularly antiseptic mouthwashes and preparations of that kind recommended for mouthwashes, are only pathological conditions existing. In the healthy mouth there is no occasion for their use, and the mouth is better off if they are left alone entirely. All that is necessary is to keep the mouth clean by the use of brushes and the other mechanical means we have, using an abrasive of some kind to remove the deposits.

Back of all this, and the thing that will do more to prevent the accumulation of deposits in the mouth, is the proper feeding of the individual. It seems to me that one of the most important things before the medical, dental, and chemical professions is the solution of the question of the proper feeding of people and how to live. We have more than the question of the kind of food we are going to eat to deal with. It is the mode of living along with the foods. Environment is another factor. We know that individual idiosyncrasies and the occupations of individuals have a great deal to do with the kind of food they can consume and require, so that in prescribing a diet for any particular individual, all of these things have to be considered.

It is, indeed, gratifying and a pleasure to me to have been here and heard this splendid presentation. I have derived much benefit from it by the thoughts the essayist has left with us regarding these very vital questions.

Dr. J. E. Hinkins. I would like to ask the essayist to give us what information he can about the use of sodium chlorid, bicarbonate of soda, and milk of magnesia as a mouthwash. I have used these for years, using the toothbrush to brush the gums as much as teeth. I have told my patients to use a half tesaspoonful of hydrate of magnesia to half a glass of water, and brush the teeth for five minutes. While I have not observed as much good from the hydrate of magnesia, I have noticed a wonderful change with bicarbonate of soda of the same strength, and that will combine readily with the saliva.

Dr. J. G. Reid. I feel I have been amply repaid for coming here and listening to this contribution. It recalls a paper which was presented before this Society some twenty years ago by Dr. Harlan on the use of mouth-washes and their detrimental effects upon the tissues of the mouth. In that paper he touched on the various products that were in use at that time, which as compared to the present number were but few. Perhaps there were five or six at that time where we have 500 at the present time. He very positively denounced the use of those drugs for the preservation of the teeth or the tissues about the teeth. His paper impressed me so deeply at the time that I could not fail to call attention to it now, and there are those present who will remember it very distinctly. I accepted very largely Dr. Harlan's conclusions in reference to this matter and have since been opposed to recommending so-called mouthwashes so extensively advertised. The manufacturers of these preparations have become fabulously rich from the sale of these products and the public think a great deal more about advertisements in papers than of the instruction from the family dentist in regard to the use of these various drugs. They will accept a newspaper advertisement as being the thing when they will not accept the advice the dentist gives them. After all, they use their own judgment, and that is true with a great many people who are sick.

I agree with Dr. Hinkins that the solution of many problems of the mouth lies within the hands of the biological chemist and the chemist, and I believe they are the men who will work out these problems more quickly possibly and with a greater degree of accuracy and determination than the bacteriologist is going to do, because I believe there are too many bacteria accumulating all the time, so that the bacteriologist cannot keep up with them. He becomes familiar with a certain group of microorganisms, and the first thing he knows there are a hundred more discovered, and he is in hot water all the time.

He does not get very far ahead in bacteriology, but chemistry is a good deal like mathematics, it is a scientific proposition, and when carried out on scientific lines we have something lasting.

Dr. Epstein (closing the discussion.) I am glad the members of this Society appreciate the work that chemists are doing in applied medicine and denistry.

In reference to the remarks made by the President (Dr. Reid) concerning the bacteriologist, the pathologist and histologist, there is no doubt that they are doing splendid work. They have taught us the structures of the human body, the size and nature of microorganisms that cause disease, and the details regarding the structure of the tissue that are diseased. All of these are important. But why do bacteria grow under certain conditions? What happens when they grow? What chemical changes take place in the tissue when diseased? What is the mechanism of these changes? It is the mechanism of chemical reaction that chemistry is trying to solve for the various industries, and especially for the medical and dental professions. Once the underlying mechanism of the change taking place is understood it is easy to find a solution for the problem.

The question of diet as applied to dental diseases and the question of mouthwashes are of vital importance to you as dentists. I have just tried to point out the path along which we are traveling and the problems that are involved. More work along this line will be required. I am not prepared to say that all mouth preparations are no good. Alkalinity and acidity of the mouth require delicate instruments to measure. It requires more than the work of the advertising manager to solve these problems for the dentist. Dentists need the cooperation of the chemist in solving these problems.

In replying to Dr. Hinkins' question, I should like to say that the continuous use of strong sodium chlorid is considered not to be good practice. Dilute sodium chlorid is present in the saliva and the chlorids aid in the digestion of starches by the ptyalin of the saliva. If a portion of the chlorids is removed from the saliva by dialysis, the digestive capacity of the saliva is impaired. Dilute bicarbonate of soda is recommended as a harmless and efficient mouthwash. It dissolves the matrix of organic material in which the inorganic insoluble calcium salts are embedded.

